# 

**Published:** 1918-09-07

**Authors:** 


September 7, 1918.] [Price Twopence

### The Hospital

The Workers' Newspaper of Administrative Medicine and Institutional Life, Administration, National Insurance and Health.

CONTENTS.

The Future of Medicine ... ... ... ... ... 481
The Subjective Influence of the Study of Medicine 482
Medical Education in War-Time ... ... ... ... 484
Specialism and Graduate Work ... ... ... 486
The Royal Naval Medical Service ... ... ... 487
The Royal Army Medical Corps ... ... ... 487
Present Conditions and Prospects.
The Public Health Services ... ... ... ... 489
Dentistry as a Profession ... ... ... ... 490
Its Drawbacks and Preparations to Qualify.
The Institutional Library ... ... ... ... 491
A Definitely-ordered Scheme of Diets—The Twentieth Edition of a Great Work—Medical Contributions to the Study of Evolution—Chemistry for Beginners—Transactions of the Incorporated Sanitary Association of Scotland, 1917.
The London Hospital Correspondence ... ... 493
Hopes for Modifications That Are Yet to Come.
A Nurses' Charter of Liberty ... ... ... ... 494
Education and Other Matters.

CONTENTS.

The Medical Curriculum ... ... ... ... 495
Qualification and Registration.
The English Conjoint Course.
The Fellowship of the Royal College of Surgeons.
London University Course.
Oxford University Course.
Cambridge University Course.
Higher Diplomas.
The Medical Student and his Work ... ... 498
The Medical Schools of the United Kingdom.
The Public Services ... ... ... ... ... 504
Graduate Institutions in London ... ... ... 506
Tropical Medicine... ... ... ... ... ... 506
Graduate Study in London Special Hospitals ... 507
The Guest Hospital, Dudley ... ... ... ... xvi
The Editor's Letter-Box ... ... ... ... xvi
The Guest Hospital, Dudley—Fulham Infirmary Nursing League.
Medical Scholarship ... ... ... ... ... xvi
The Book Market ... ... ... ... ... xvi
The Attractions of Dispensing as a Profession ... xvi

